# Emerging roles of KIR2DL4 in cancer immunotherapy

**DOI:** 10.1007/s12282-025-01738-y

**Published:** 2025-06-23

**Authors:** Weimiao Li, Guoxu Zheng, Shuqun Zhang

**Affiliations:** 1 The Comprehensive Breast Care Center, The Second Affiliated Hospital of Xi’an Jiaotong University, No.157, Xiwu Road, Xi’an, 710004 China; 2 State Key Laboratory of Holistic Integrative Management of Gastrointestinal Cancers and Department of Immunology, Fourth Military Medical University, No.169, Changle West Road, Xi’an, 710032 China

**Keywords:** KIR2DL4, Immune checkpoint, Cancer immunotherapy, NK cells

## Abstract

Killer-cell immunoglobulin-like receptor 2DL4 (KIR2DL4), a member of the killer cell immunoglobulin-like receptors (KIRs) family, plays an important role in the regulation of the immune system, which is expressed primarily on natural killer (NK) cells. Human leucocyte antigen-G (HLA-G), a non-classical major histocompatibility complex (MHC) class I molecule, is the only known ligand of KIR2DL4. Accumulating evidence has shown that KIR2DL4 has emerged as a potential target for enhancing the antitumor immune response. Elevated expression of KIR2DL4 has been observed in certain tumor types, including melanoma, lung cancer, and ovarian cancer, indicating its role in tumor evasion. Our previous study had shown that blockade of KIR2DL4 interaction in NK cells can re-sensitize breast cancer to trastuzumab treatment, which indicated that KIR2DL4 was a pivotal immune checkpoint of NK cells. Currently, there are several therapeutic approaches targeting KIR in cancer immunotherapy. However, there are no efficient cancer immunotherapy strategy targeting KIR2DL4. In this review, we aim to summarize and discuss the potential role of KIR2DL4 as a target for cancer immunotherapy. A better understanding of KIR2DL4 might be helpful to develop effective KIR2DL4-targeted therapies, which could provide new treatment options for cancer patients.

## Introduction

Cancer immunotherapy has emerged as a transformative approach in oncology, offering a new paradigm for treating various malignancies [1]. It encompasses a range of strategies designed to stimulate the immune system to target and eliminate cancer cells [2]. This approach offers targeted and personalized therapy with durable responses and fewer side effects than traditional chemotherapy or radiotherapy. Cancer immunotherapy consists of four categories, including immune checkpoint blockade, tumor vaccines, adoptive cell immunotherapy, and oncolytic viruses [3]. Immunotherapy has led to remarkable improvements in survival for patients with melanoma, lung cancer, and several hematological malignancies [4–6]. It is also used in combination with other therapies, such as chemotherapy or radiation, to enhance their effectiveness [7, 8]. While immunotherapy has transformed cancer treatment, it is not effective in all patients. Predicting which patients will respond to immunotherapy remains a challenge. Future research will focus on identifying biomarkers that can predict immunotherapy response and on developing new immunotherapy strategies to target a broader range of cancers.

KIR2DL4, also known as killer cell immunoglobulin-like receptor 2DL4, is a member of the inhibitory killer cell immunoglobulin-like receptors (KIRs) family, which plays a crucial role in regulating the immune system [9]. HLA-G, a non-classical MHC class I molecule, is the only known ligand of KIR2DL4 [10]. By binding to HLA-G, KIR2DL4 can transmit inhibitory signal to immune cells, suppressing their activation and proliferation [9]. This interaction is crucial for maintaining immune homeostasis. KIR2DL4 plays a vital role in protecting the fetus from maternal immune attack. KIR2DL4 on maternal NK cells can recognize HLA-G expressed by the trophoblasts, which allows the fetus to evade immune surveillance via resulting in the suppression of NK cell activation and the promotion of a tolerogenic immune environment [10]. In addition to its role in pregnancy, KIR2DL4 also plays a role in anti-infective immune response. It has been shown to regulate NK cell and T cell responses against viral infections [11]. By understanding the function and regulation of KIR2DL4, we can gain insights into the immune system and develop more effective strategies for treating immune-related diseases and disorders.

Accumulated evidences have shown that KIR2DL4 plays an important role in tumor immune escape [12]. KIR2DL4 can interact with its ligand HLA-G, which is often overexpressed in tumor cells. This interaction further inhibits the antitumor activity of immune cells, providing a mechanism for tumor evasion from immune surveillance. Our prior research demonstrated that inhibiting the interaction of KIR2DL4 in NK cells can enhance the efficacy of trastuzumab treatment in breast cancer, suggesting that KIR2DL4 plays a crucial role as an immune checkpoint in NK cells [13, 14]. According to this study, blockade of KIR2DL4/HLA-G signaling using specific antibodies or other immunomodulatory agents, might be possible to enhance the antitumor activity of immune cells and improve the efficacy of tumor immunotherapy. In this review, we summarized and discussed the pivotal role of KIR2DL4 in tumor immunotherapy by regulating the immune response against tumor cells. Deeply understanding the functions and interactions of KIR2DL4 may lead to the development of more effective immunotherapy strategies that harness the immune system against cancer cells.

## The structure of KIR2DL4

**Fig. 1:**
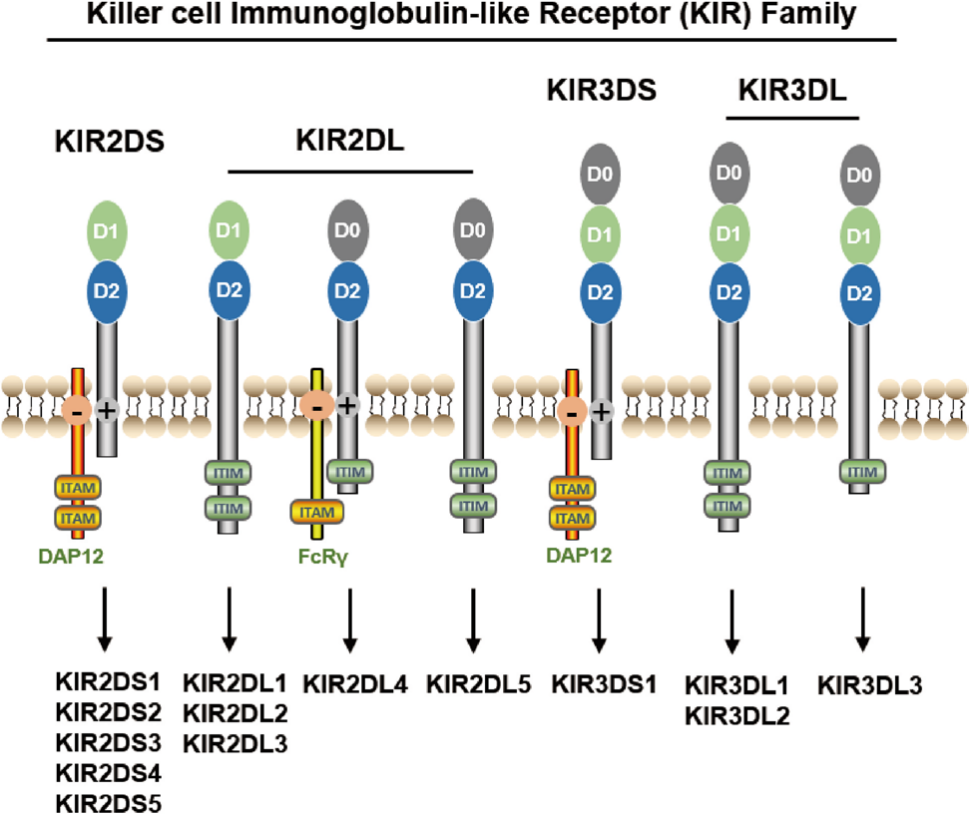
A schematic structure of killer cell immunoglobulin-like receptor family

KIR2DL4 is a member of the killer cell immunoglobulin-like receptors (KIRs) family [9]. The *KIR* genes are situated on chromosome 19q13.4, each encompassing a range of 10,000–15,000 bps and being interspersed by 1,000 bp. *KIR* genes are comprised of a total of nine exons, with specific exons encoding distinct structural components: exons 1 and 2 encode the leader peptide, exons 3–5 encode the extracellular domains, exon 6 encodes the stem structure, exon 7 encodes the transmembrane region, and exons 8–9 encode the intracellular region [15]. The structure of KIRs is relatively conserved and consists of several distinct domains (Fig. 1). In general, KIRs are type I transmembrane glycoproteins that are anchored in the cell membrane by a single transmembrane domain. They are composed of an extracellular domain, a transmembrane domain, and a cytoplasmic domain. The extracellular domain of KIRs is composed of immunoglobulin-like folds and is responsible for binding to MHC class I molecules on the surface of target cells. This domain typically contains two (2D) or three immunoglobulin-like domains (3D), depending on the KIR subtype [15]. The binding specificity of KIRs for MHC class I molecules is determined by the amino acid sequences within these immunoglobulin-like domains. The transmembrane domain of KIRs is a hydrophobic region that anchors the protein in the cell membrane. It consists of approximately 20–25 amino acids and is responsible for attaching the KIR to the plasma membrane [15]. The cytoplasmic domain of KIRs is located within the cell and plays a crucial role in signal transduction. It contains either a long (L) or a short (S) cytoplasmic tail, depending on the KIR subtype [15]. KIRs with long cytoplasmic tails (L-KIRs) contain immunoreceptor tyrosine-based inhibitory motifs (ITIMs) that become phosphorylated upon ligand binding. This phosphorylation can recruit phosphatases, such as SHP-1, which dephosphorylate activating receptors and thereby downregulate immune responses. In contrast, KIRs with short cytoplasmic tails (S-KIRs) lack ITIMs and instead associate with TYRO protein tyrosine kinase-binding protein (TYROBP), such as DAP12 and FcRγ, which contain immunoreceptor tyrosine-based activation motifs (ITAMs) to transduce activating signals. In addition to these three domains, KIRs also contain a leader sequence at the N-terminus that is responsible for directing the protein to the endoplasmic reticulum for proper folding and trafficking to the cell surface [10].

A partial deletion at the end of exon 7 in KIR2DL4 transcripts was observed, which was facilitated by an alternative 5’ splice site. The termination of exon 7 in KIR2DL4 is demarcated by a poly-adenine sequence that can consist of either nine (9A) or ten (10A) nucleotides [15]. The presence of a premature stop codon following exon 7 in the 9 A KIR2DL4 alleles indicates the lack of a cytoplasmic tail, resulting in the loss of their inhibitory capacity. Conversely, the 10 A KIR2DL4 transcripts encode a full receptor, which includes a cytoplasmic tail containing a single ITIM [15]. KIR2DL4 is structurally unique among the KIR family due to its dual role as both an activating and inhibitory receptor. KIR2DL4 is composed of several distinct domains that contribute to its function and interaction with its ligand. At the amino terminus, KIR2DL4 features a leader sequence that directs its trafficking to the cell surface. Following the leader sequence is the extracellular domain, which is further divided into two immunoglobulin-like folds, known as domains 0 (D0) and 2(D2) [9]. These domains are responsible for binding to MHC class I molecules and other ligands, mediating the inhibitory or activating signals transmitted by KIR2DL4. Within the extracellular domain, KIR2DL4 exhibits a high degree of polymorphism, particularly in domain 0 [16]. This polymorphism affects the binding affinity and specificity of KIR2DL4 for its ligands, contributing to the diverse functional outcomes observed upon engagement. The transmembrane domain of KIR2DL4 anchors the protein to the cell membrane, facilitating its interaction with intracellular signaling molecules. The cytoplasmic tail of KIR2DL4 is relatively short and lacks a signaling motif, suggesting that it may rely on other associated molecules or adaptor proteins for signal transduction [9]. In contrast to the typical KIR2DL receptors, KIR2DL4 is distinguished by the presence of a single ITIM in its cytoplasmic tail rather than the usual two, as well as a positively charged arginine in its transmembrane region that facilitates the recruitment of activation adaptors (Fig. 1). KIR2DL4 has both an activating and inhibitory potential, which can influence the cytotoxic activity of NK cells. The balance between these functions can determine the immune response to target cells.

## The expression of KIR2DL4 and HLA-G

KIR2DL4, also known as CD158d, exhibits predominant expression on NK cells, with evidence suggesting its presence on select subsets of T cells. The expression levels of KIR2DL4 are subject to interindividual variation and can be modulated by various factors such as genetic polymorphisms, and cytokines. Accumulated evidence has shown KIRs exhibit high levels of polymorphism and contribute to the wide range of diversity within the human NK cell repertoire through three key attributes [17]. Firstly, individuals have the potential to inherit distinct haplotypes of the 14 KIR family member genes, resulting in diverse gene content that may consist predominantly of inhibitory KIRs (haplotype A) or a more heterogeneous repertoire with a higher proportion of activating KIRs (haplotype B) [18]. Furthermore, individual KIRs have the ability to identify specific subsets of classical MHC-I ligands. Additionally, the expression of KIR genes on NK cells is random, resulting in the generation of a varied repertoire. In contrast to other KIR2DL receptors, KIR2DL4 is unique in being expressed by all NK cells and present in all individuals and comprises the framework genes of the locus and is grouped into all KIR haplotypes [19–21]. Goodridge *et.al* found that the 10 A KIR2DL4 allele encodes a full-length receptor with significant membrane expression, while the 9 A KIR2DL4 allele results in a truncated protein that lacks the inhibitory motif. The study demonstrates that individuals with at least one 10 A allele show cell surface expression of KIR2DL4, primarily on the CD56^bright^ NK cell subset [22]. Additionally, Goodridge *et.al* reported that individuals with the 10 A KIR2DL4 allele may lack detectable KIR2DL4 on resting CD56^bright^ NK cells due to the excision of the D0 domain and the 9 A KIR2DL4 allele can produce a secreted KIR2DL4 receptor [23]. All these studies indicated that KIR2DL4 can be detected on all NK cell subset. Our previous study has shown that KIR2DL4 facilitates antibody-dependent cell-mediated cytotoxicity (ADCC) and establishes a regulatory relationship with the interferon-γ (IFN-γ) production pathway. This pathway involves IFN-γ inducing the upregulation of KIR2DL4 through JAK2/STAT1 signaling, followed by KIR2DL4 working in conjunction with the Fcγ receptor to enhance IFN-γ secretion by NK cells [13]. Akiko *et.al* reported that KIR2DL4 expressed on the cell surface can be up-regulated by IL-2 [24].

HLA-G, a non-classical MHC class I molecule, is the only known ligand of KIR2DL4 [25]. The *HLA-G* gene consists of 8 exons and 7 introns, with most full-length HLA-G transcripts typically containing 7 exons due to the removal of exon 7 through splicing. In comparison to classical HLA class I molecules, HLA-G is relatively short, with a total length of approximately 340 amino acids. The signal peptide is encoded by exon 1, while the three extracellular domains (α1-α3) are translated from exons 2, 3, and 4, respectively. The transmembrane region is generated by exon 5, and the intracellular region is generated by exon 6. Accumulating evidence has demonstrated the existence of seven isoforms of HLA-G, each characterized by a unique molecular structure resulting from distinct transcript splicing events [9]. Among these isoforms, HLA-G1 exhibits both membrane-binding and soluble forms, featuring three extracellular domains (α1-α3), while HLA-G2, HLA-G3, and HLA-G4 are exclusively membrane-binding isoforms. Specifically, HLA-G2 possesses two extracellular domains (α1 and α3), HLA-G3 contains only the α1 domain, and HLA-G4 displays two extracellular domains (α1 and α2). Conversely, HLA-G5, HLA-G6, and HLA-G7 are classified as soluble isoforms, with HLA-G5 presenting three extracellular domains (α1-α3), HLA-G6 featuring two extracellular domains (α1 and α3), and HLA-G7 having an α1 domain. In contrast to the classical MHC class I molecule, The D0 domain of KIR2DL4 interacts with the α1 domain of HLA-G within the peptide binding cleft. Nevertheless, the peptide is situated at a greater depth within the binding cleft compared to the location where KIR2DL4 binds, indicating that the peptide does not play a role in the binding interaction between KIR2DL4 and HLA-G [26–28]. Despite the predominant expression of HLA-G in the fetal cytotrophoblast, its physiological presence has been documented in adult stem cells, progenitor cells, somatic cells in immune-privileged tissues, and certain immune cells [29–31]. Additionally, ectopic expression of HLA-G has been observed in various pathological states, such as tumor [32].

## Role of KIR2DL4 in cancer immunotherapy

KIR2DL4 stands out among KIRs for its distinct capacity to interact with HLA-G, a ligand frequently overexpressed on tumor cells as a means of evading immune surveillance [33, 34]. This interaction has the potential to impede NK cell-mediated cytotoxicity, thereby facilitating uncontrolled tumor progression [35]. Consequently, the investigation of the targeting KIR2DL4 therapy has garnered significant attention in the realm of research, as it presents promising opportunities for the advancement of novel immunotherapeutic approaches aimed at bolstering anti-tumor immune responses. He *et.al* found that 10 candidate hub genes including KIR2DL4 exhibit a significant correlation with immune infiltration, which has successfully established a comprehensive immune network and systematically elucidated the molecular mechanisms associated with endometriosis [36]. Recent study has found a novel comprehensive index of aging and immune (CIAI) signature comprising 7 genes, including KIR2DL4, in which patients with melanoma in the high-CIAI group exhibited significantly shorter overall survival (OS), disease-specific survival (DSS), and progression-free interval (PFI), suggesting that the CIAI model can serve as an independent prognostic index. Additionally, their analysis revealed potential correlations between the CIAI score and immune scores, estimate score, immune cell infiltration levels, tumor microenvironment characteristics, immunotherapy response, and drug sensitivity [37]. Multiple studies have demonstrated that the expression levels of KIR2DL4 may serve as a potential prognostic marker for melanoma, indicating the likelihood of disease aggressiveness [38–41].

Breast cancer frequently exploits immune checkpoints to evade surveillance. Ozturk *et.al* found that the frame genes of KIR2DL4 were found in all breast cancer patients [42]. In 2015, Ueshima and their colleagues found that the KIR2DL4 on human mast cells facilitates HLA-G-expressing breast cancer invasion and the subsequent metastasis [43]. Our previous study has shown that the interaction between HLA-G and KIR2DL4 on NK cells inhibits trastuzumab-induced ADCC, but blocking this interaction can enhance the effectiveness of trastuzumab in combating tumors in vivo. Conversely, when not bound by HLA-G, KIR2DL4 can stimulate ADCC and establish a regulatory loop with the IFN-γ production pathway through JAK2/STAT1 signaling in NK cells. Furthermore, the presence of paracrine TGF-β and IFN-γ in the breast cancer microenvironment can induce the expression of PD-1/PD-L1 on NK cells and tumor cells, potentially exacerbating immunosuppression through enhanced intercellular signaling. These findings underscore the potential clinical implications of targeting HLA-G/KIR2DL4 interactions to enhance antitumor immune responses [13]. All these studies showed that KIR2DL4 plays an important role in cancer immunotherapy and targeting KIR2DL4 might be an effective approach.

Due to the special structure, KIR2DL4 can deliver both activating and inhibitory signals. Based on this, there are few effective targeting KIR2DL4 treatment regimens have been developed. However, there are a series of KIRs based immunotherapy strategies that can provide new insights into future cancer immunotherapies targeting KIR2DL4, such as immune checkpoint blockade (ICB) therapy, adoptive cell transfer (ACT) therapy and small-molecule inhibitors [44]. The cytotoxic activity of NK cells against healthy host cells is primarily inhibited by the interaction between inhibitory KIRs and MHC class I molecules. Previous findings suggest that tumor cells evade NK cell-mediated killing by increasing the expression of MHC class I molecules. Consequently, researchers have focused on inhibitory KIRs as a key regulatory mechanism in NK cell function and have explored the use of specific antibodies to block these receptors in clinical trials [44]. IPH2101 and Lirilumab (IPH2102), which are considered the most suitable antibodies, along with anti-KIR 1-7F9 IPH2101 (previously known as 1-7F9), the initial agent in this category, have been utilized to target KIR2DL1/2/3 to enhance NK cell cytotoxicity [45, 46]. Ren et.al elucidated the functional mechanisms by which KIR2DL5 mediates NK cell immune evasion, demonstrated the potential therapeutic efficacy of blocking the KIR2DL5/PVR axis in human cancers, and offered insight into the underlying mechanism for the lack of clinical success of anti-TIGIT therapies [47]. All these studies suggested that blockade of KIR2DL4-HLA-G interaction by using an agonistic antibody targeting KIR2DL4 might have therapeutic potential. Series studies have found that KIRs play a critical role in controlling NK cell function. Strategies to improve CAR-NK cell function include targeting inhibitory KIR to prevent trogocytosis-mediated fratricide and activating KIR-rich NK cell donors may be beneficial for allogeneic NK cell therapy, which indicates that targeting KIR has the potential to enhance patient outcomes and efficacy of CAR-NK cell therapy [48, 49]. These studies suggested that targeting KIR2DL4 by CAR-NK might switch inhibitory signals to ablate HLA-G positive tumor cells. As previously stated, SHP-1 and SHP-2 play crucial roles as primary mediators in the inhibitory signaling cascade. The interaction between inhibitory KIR and MHC class I molecules can result in the dephosphorylation of vav1 downstream of activating receptors by SHP-1. Subsequently, this process leads to the dephosphorylation of ERK and STAT3, ultimately suppressing NK cell cytotoxicity against normal cells [50, 51]. Hence, targeting SHP-1 and SHP-2 as pivotal checkpoints in cancer immunotherapy to enhance NK cell activity within tumor microenvironments is a viable strategy. Experimental studies utilizing SHP-1 and SHP-2 inhibitor have shown promising results in the realm of cancer therapeutics [52–54]. KIR2DL4 can also recruit SHP-1 and SHP-2 to antagonize kinase-dependent activation cascades via ITIM signaling motif, which indicates that targeting SHP-1 and SHP-2 might be an efficient approach to retain the active potential of KIR2DL4. Recent studies have found that KIR2DL4 might be a tumor suppressor via its activating potential in breast cancer [55], which is consistent with the results in our previous study [13]. In general, the development of therapies could involve the use of monoclonal antibodies that specifically target KIR2DL4 or its interaction with HLA-G. In the case of therapies that target the interaction between KIR2DL4 and HLA-G, there is a risk of disrupting normal immune functions that rely on this interaction. Since KIR2DL4 is also involved in normal physiological processes, such as in pregnancy related immune tolerance, targeting it could potentially lead to unintended consequences in the immune system. Additionally, if the therapy affects NK cell function too profoundly, it could compromise the body’s natural defense against infections and other malignancies, highlighting the need for careful evaluation of potential side effects in the development of KIR2DL4 targeted therapies.

## Conclusion

The immune system has been identified as a significant factor in the development and advancement of cancer. Various immunotherapy methods, such as targeting tumor antigens with antibodies, immune checkpoint blockade, and adoptive cell transfer (ACT) therapy, have been explored for the treatment of cancer patients. Recent research has indicated that combining traditional chemotherapy or radiotherapy with immunotherapy can lead to better outcomes for cancer patients [56, 57]. The treatment approaches for cancer have evolved from cytotoxic therapy to enhancing anti-tumor immune responses. However, a limited proportion of cancer patients are able to derive advantages from immunotherapy at present. The efficacy of immunotherapy is hindered by the comparatively low response rate and the presence of an immunosuppressive tumor microenvironment even among those who do respond. The dysregulated expression of immune checkpoint molecules is identified as the primary factor contributing to immune evasion. Consequently, immune checkpoint blockade emerges as a crucial strategy for counteracting the immunosuppressive conditions within the tumor microenvironment. Combining KIR2DL4 targeted therapies with immune checkpoint inhibitors that target other molecules, such as PD-1/PD-L1, could also have synergistic effects. By simultaneously targeting different immune regulatory pathways, it may be possible to enhance the antitumor immune response and improve patient outcomes. Prospective studies should carefully evaluate the safety and efficacy of these combination therapies, taking into account factors such as potential drug-drug interactions and additive side effects.

This review provides an overview of the distinctive structural characteristics of KIR2DL4, followed by an examination of its expression within the immune microenvironment of tumor and the potential utility of targeting KIR2DL4 in cancer immunotherapy. KIR2DL4 is the only KIR receptor known to interact with HLA-G. It possesses an ITIM domain within its cytoplasmic tail and a positively charged arginine in its transmembrane region, facilitating the recruitment of activation adaptors containing an ITAM domain. Due to its unique structural features, KIR2DL4 plays a crucial role in orchestrating a complex interplay between immune checkpoint signaling and cytokine responses within the tumor microenvironment. The engagement of HLA-G by KIR2DL4 ultimately determines the efficacy and outcome of immunotherapeutic interventions. The long-term outlook for KIR2DL4 targeted cancer immunotherapy is promising but also faces challenges. If the challenges related to specificity, efficacy, and side effects can be addressed, KIR2DL4 targeted therapies could become an important part of the cancer treatment arsenal. In the long term, these therapies could potentially be used in combination with other emerging immunotherapies and targeted drugs to provide more personalized treatment options for cancer patients. As our understanding of the role of KIR2DL4 in the tumor immune microenvironment continues to grow, more precise and effective therapies can be developed. Additionally, with the advancement of technologies for KIR2DL4 modulation, such as gene editing and novel imaging techniques, the future of KIR2DL4-targeted cancer immunotherapy looks promising. However, continued research, well-designed clinical trials, and careful consideration of ethical and safety aspects are essential for realizing the full potential of these therapies in improving cancer patient survival and quality of life.

## Data Availability

The data are available from the corresponding author on reasonable request.

## References

[1] Lu Q, Kou D, Lou S, et al. Nanoparticles in tumor microenvironment remodeling and cancer immunotherapy. J Hematol Oncol. 2024;17(1):16. 38566199 10.1186/s13045-024-01535-8 PMC10986145

[2] G.R. Khosravi, S. Mostafavi, S. Bastan, et al., *Immunologic tumor microenvironment modulators for turning cold tumors hot.* Cancer Commun (Lond), 2024. 10.1002/cac2.12539 PMC11110955 38551889

[3] Marin-Acevedo JA, Soyano AE, Dholaria B, et al. Cancer immunotherapy beyond immune checkpoint inhibitors. J Hematol Oncol. 2018;11(1):8. 29329556 10.1186/s13045-017-0552-6 PMC5767051

[4] Sun W, Zhu Y, Zou Z, et al. An advanced comprehensive muti-cell-type-specific model for predicting anti-PD-1 therapeutic effect in melanoma. Theranostics. 2024;14(5):2127–50. 38505619 10.7150/thno.91626 PMC10945348

[5] Tsai YT, Schlom J, Donahue RN. Blood-based biomarkers in patients with non-small cell lung cancer treated with immune checkpoint blockade. J Exp Clin Cancer Res. 2024;43(1):82. 38493133 10.1186/s13046-024-02969-1 PMC10944611

[6] Dagher OK, Posey AD Jr. Forks in the road for CAR T and CAR NK cell cancer therapies. Nat Immunol. 2023;24(12):1994–2007. 38012406 10.1038/s41590-023-01659-y PMC12798859

[7] Y. Han, M. Jiang, Y. Sun, et al., *Efficient chemo-immunotherapy leveraging minimalist electrostatic complex nanoparticle as "in situ" vaccine integrated tumor ICD and immunoagonist.* J Adv Res, 2024. 10.1016/j.jare.2024.03.010 PMC11954839 38499244

[8] Y. Tsukita, T. Tozuka, K. Kushiro, et al., *Immunotherapy or Chemoimmunotherapy in Older Adults With Advanced Non-Small Cell Lung Cancer.* JAMA Oncol, 2024. 10.1001/jamaoncol.2023.6277 PMC10921348 38451530

[9] Zheng G, Jia L, Yang AG. Roles of HLA-G/KIR2DL4 in Breast Cancer Immune Microenvironment. Front Immunol. 2022;13: 791975. 35185887 10.3389/fimmu.2022.791975 PMC8850630

[10] Rajagopalan S, Long EO. A human histocompatibility leukocyte antigen (HLA)-G-specific receptor expressed on all natural killer cells. J Exp Med. 1999;189(7):1093–100. 10190900 10.1084/jem.189.7.1093 PMC2193010

[11] Feng Z, Huang P, Zhang J, et al. KIR2DL4/HLA-G polymorphisms were associated with HCV infection susceptibility among Chinese high-risk population. J Med Virol. 2023;95(3): e28645. 36890645 10.1002/jmv.28645

[12] LeMaoult J, Zafaranloo K, Le Danff C, et al. HLA-G up-regulates ILT2, ILT3, ILT4, and KIR2DL4 in antigen presenting cells, NK cells, and T cells. Faseb j. 2005;19(6):662–4. 15670976 10.1096/fj.04-1617fje

[13] Zheng G, Guo Z, Li W, et al. Interaction between HLA-G and NK cell receptor KIR2DL4 orchestrates HER2-positive breast cancer resistance to trastuzumab. Signal Transduct Target Ther. 2021;6(1):236. 34158475 10.1038/s41392-021-00629-w PMC8219715

[14] Guo Z, Zhang R, Yang AG, et al. Diversity of immune checkpoints in cancer immunotherapy. Front Immunol. 2023;14:1121285. 36960057 10.3389/fimmu.2023.1121285 PMC10027905

[15] Bruijnesteijn J, van der Wiel MKH, de Groot N, et al. Extensive Alternative Splicing of KIR Transcripts. Front Immunol. 2018;9:2846. 30564240 10.3389/fimmu.2018.02846 PMC6288254

[16] Buhler S, Di Cristofaro J, Frassati C, et al. High levels of molecular polymorphism at the KIR2DL4 locus in French and Congolese populations: impact for anthropology and clinical studies. Hum Immunol. 2009;70(11):953–9. 19679155 10.1016/j.humimm.2009.08.002

[17] Campbell KS, Purdy AK. Structure/function of human killer cell immunoglobulin-like receptors: lessons from polymorphisms, evolution, crystal structures and mutations. Immunology. 2011;132(3):315–25. 21214544 10.1111/j.1365-2567.2010.03398.x PMC3044898

[18] Maskalenko NA, Zhigarev D, Campbell KS. Harnessing natural killer cells for cancer immunotherapy: dispatching the first responders. Nat Rev Drug Discov. 2022;21(8):559–77. 35314852 10.1038/s41573-022-00413-7 PMC10019065

[19] Tukwasibwe S, Nakimuli A, Traherne J, et al. Variations in killer-cell immunoglobulin-like receptor and human leukocyte antigen genes and immunity to malaria. Cell Mol Immunol. 2020;17(8):799–806. 32541835 10.1038/s41423-020-0482-z PMC7294524

[20] Djaoud Z, Parham P. HLAs, TCRs, and KIRs, a Triumvirate of Human Cell-Mediated Immunity. Annu Rev Biochem. 2020;89:717–39. 32569519 10.1146/annurev-biochem-011520-102754

[21] Manser AR, Weinhold S, Uhrberg M. Human KIR repertoires: shaped by genetic diversity and evolution. Immunol Rev. 2015;267(1):178–96. 26284478 10.1111/imr.12316

[22] Goodridge JP, Witt CS, Christiansen FT, et al. KIR2DL4 (CD158d) genotype influences expression and function in NK cells. J Immunol. 2003;171(4):1768–74. 12902476 10.4049/jimmunol.171.4.1768

[23] Goodridge JP, Lathbury LJ, Steiner NK, et al. Three common alleles of KIR2DL4 (CD158d) encode constitutively expressed, inducible and secreted receptors in NK cells. Eur J Immunol. 2007;37(1):199–211. 17171757 10.1002/eji.200636316

[24] Kikuchi-Maki A, Yusa S, Catina TL, et al. KIR2DL4 is an IL-2-regulated NK cell receptor that exhibits limited expression in humans but triggers strong IFN-gamma production. J Immunol. 2003;171(7):3415–25. 14500636 10.4049/jimmunol.171.7.3415

[25] Wang S, Wang J, Xia Y, et al. Harnessing the potential of HLA-G in cancer therapy: advances, challenges, and prospects. J Transl Med. 2024;22(1):130. 38310272 10.1186/s12967-024-04938-w PMC10838004

[26] Rouas-Freiss N, Moreau P, LeMaoult J, et al. Role of the HLA-G immune checkpoint molecule in pregnancy. Hum Immunol. 2021;82(5):353–61. 33745758 10.1016/j.humimm.2021.01.003

[27] Mbiribindi B, Mukherjee S, Wellington D, et al. Spatial Clustering of Receptors and Signaling Molecules Regulates NK Cell Response to Peptide Repertoire Changes. Front Immunol. 2019;10:605. 31024524 10.3389/fimmu.2019.00605 PMC6460049

[28] J.V.D. Attia, C.E. Dessens, R. van de Water, et al., *The Molecular and Functional Characteristics of HLA-G and the Interaction with Its Receptors: Where to Intervene for Cancer Immunotherapy?* Int J Mol Sci, 2020. **21**(22). 10.3390/ijms21228678 PMC7698525 33213057

[29] Contini P, Murdaca G, Puppo F, et al. HLA-G Expressing Immune Cells in Immune Mediated Diseases. Front Immunol. 2020;11:1613. 32983083 10.3389/fimmu.2020.01613 PMC7484697

[30] Loustau M, Anna F, Dréan R, et al. HLA-G Neo-Expression on Tumors Front Immunol. 2020;11:1685. 32922387 10.3389/fimmu.2020.01685 PMC7456902

[31] Xu X, Zhou Y, Wei H. Roles of HLA-G in the Maternal-Fetal Immune Microenvironment. Front Immunol. 2020;11: 592010. 33193435 10.3389/fimmu.2020.592010 PMC7642459

[32] Xu HH, Gan J, Xu DP, et al. Comprehensive Transcriptomic Analysis Reveals the Role of the Immune Checkpoint HLA-G Molecule in Cancers. Front Immunol. 2021;12: 614773. 34276642 10.3389/fimmu.2021.614773 PMC8281136

[33] Jing R, Bai S, Zhang P, et al. IDO-1 impairs antitumor immunity of natural killer cells in triple-negative breast cancer via up-regulation of HLA-G. Breast Cancer. 2024;31(1):135–47. 37981615 10.1007/s12282-023-01522-w PMC10764509

[34] F. Morandi and I. Airoldi, *HLA-G and Other Immune Checkpoint Molecules as Targets for Novel Combined Immunotherapies.* Int J Mol Sci, 2022. **23**(6). 10.3390/ijms23062925 PMC8948858 35328349

[35] Nersesian S, Carter EB, Lee SN, et al. Killer instincts: natural killer cells as multifactorial cancer immunotherapy. Front Immunol. 2023;14:1269614. 38090565 10.3389/fimmu.2023.1269614 PMC10715270

[36] Lv SJ, Sun JN, Gan L, et al. Identification of molecular subtypes and immune infiltration in endometriosis: a novel bioinformatics analysis and In vitro validation. Front Immunol. 2023;14:1130738. 37662927 10.3389/fimmu.2023.1130738 PMC10471803

[37] Lv W, Zhan Y, Tan Y, et al. A combined aging and immune prognostic signature predict prognosis and responsiveness to immunotherapy in melanoma. Front Pharmacol. 2022;13: 943944. 36034849 10.3389/fphar.2022.943944 PMC9402914

[38] Mao R, Ren Z, Yang F, et al. Clinical significance and immune landscape of KIR2DL4 and the senescence-based signature in cutaneous melanoma. Cancer Sci. 2022;113(11):3947–59. 35848898 10.1111/cas.15499 PMC9633315

[39] He Z, Chen M, Luo Z. Identification of immune-related genes and integrated analysis of immune-cell infiltration in melanoma. Aging (Albany NY). 2024;16(1):911–27. 38217549 10.18632/aging.205427 PMC10817386

[40] Qu Y, Zhang S, Zhang Y, et al. Identification of immune-related genes with prognostic significance in the microenvironment of cutaneous melanoma. Virchows Arch. 2021;478(5):943–59. 33179141 10.1007/s00428-020-02948-9

[41] Zhou S, Sun Y, Chen T, et al. The Landscape of the Tumor Microenvironment in Skin Cutaneous Melanoma Reveals a Prognostic and Immunotherapeutically Relevant Gene Signature. Front Cell Dev Biol. 2021;9: 739594. 34660598 10.3389/fcell.2021.739594 PMC8517264

[42] Ozturk OG, Gun FD, Polat G. Killer cell immunoglobulin-like receptor genes in patients with breast cancer. Med Oncol. 2012;29(2):511–5. 21479698 10.1007/s12032-011-9932-x

[43] Ueshima C, Kataoka TR, Hirata M, et al. The Killer Cell Ig-like Receptor 2DL4 Expression in Human Mast Cells and Its Potential Role in Breast Cancer Invasion. Cancer Immunol Res. 2015;3(8):871–80. 25735953 10.1158/2326-6066.CIR-14-0199

[44] K. Dizaji Asl, K. Velaei, A. Rafat, et al., *The role of KIR positive NK cells in diseases and its importance in clinical intervention.* Int Immunopharmacol, 2021. **92**: p. 107361. 10.1016/j.intimp.2020.107361 33429335

[45] Romagné F, André P, Spee P, et al. Preclinical characterization of 1–7F9, a novel human anti-KIR receptor therapeutic antibody that augments natural killer-mediated killing of tumor cells. Blood. 2009;114(13):2667–77. 19553639 10.1182/blood-2009-02-206532 PMC2756126

[46] R. Maeoka, T. Nakazawa, R. Matsuda, et al., *Therapeutic Anti-KIR Antibody of 1–7F9 Attenuates the Antitumor Effects of Expanded and Activated Human Primary Natural Killer Cells on In Vitro Glioblastoma-like Cells and Orthotopic Tumors Derived Therefrom.* Int J Mol Sci, 2023. **24**(18). 10.3390/ijms241814183 PMC10531877 37762486

[47] X. Ren, M. Peng, P. Xing, et al., *Blockade of the immunosuppressive KIR2DL5/PVR pathway elicits potent human NK cell-mediated antitumor immunity.* J Clin Invest, 2022. **132**(22). 10.1172/JCI163620 PMC9663162 36377656

[48] Graham LV, Fisher JG, Khakoo SI, et al. Targeting KIR as a novel approach to improve CAR-NK cell function. J Transl Genet Genom. 2023;7:230–5. 38229912 10.20517/jtgg.2023.25 PMC7615527

[49] Li Y, Basar R, Wang G, et al. KIR-based inhibitory CARs overcome CAR-NK cell trogocytosis-mediated fratricide and tumor escape. Nat Med. 2022;28(10):2133–44. 36175679 10.1038/s41591-022-02003-x PMC9942695

[50] Stebbins CC, Watzl C, Billadeau DD, et al. Vav1 dephosphorylation by the tyrosine phosphatase SHP-1 as a mechanism for inhibition of cellular cytotoxicity. Mol Cell Biol. 2003;23(17):6291–9. 12917349 10.1128/MCB.23.17.6291-6299.2003 PMC180957

[51] Teng R, Wang Y, Lv N, et al. Hypoxia Impairs NK Cell Cytotoxicity through SHP-1-Mediated Attenuation of STAT3 and ERK Signaling Pathways. J Immunol Res. 2020;2020:4598476. 33123602 10.1155/2020/4598476 PMC7584946

[52] Chen YN, LaMarche MJ, Chan HM, et al. Allosteric inhibition of SHP2 phosphatase inhibits cancers driven by receptor tyrosine kinases. Nature. 2016;535(7610):148–52. 27362227 10.1038/nature18621

[53] Zhao M, Guo W, Wu Y, et al. SHP2 inhibition triggers anti-tumor immunity and synergizes with PD-1 blockade. Acta Pharm Sin B. 2019;9(2):304–15. 30972278 10.1016/j.apsb.2018.08.009 PMC6437555

[54] Naing A, Reuben JM, Camacho LH, et al. Phase I Dose Escalation Study of Sodium Stibogluconate (SSG), a Protein Tyrosine Phosphatase Inhibitor, Combined with Interferon Alpha for Patients with Solid Tumors. J Cancer. 2011;2:81–9. 21326629 10.7150/jca.2.81 PMC3039225

[55] Kilic N, Dastouri M, Kandemir I, et al. The effects of KIR2DL4 stimulated NK-92 cells on the apoptotic pathways of HER2 + /HER-breast cancer cells. Med Oncol. 2023;40(5):139. 37027073 10.1007/s12032-023-02009-6

[56] Zhang X, Cai X, Yan C. Opportunities and challenges in combining immunotherapy and radiotherapy in esophageal cancer. J Cancer Res Clin Oncol. 2023;149(20):18253–70. 37985502 10.1007/s00432-023-05499-z PMC10725359

[57] Zhang Z, Liu X, Chen D, et al. Radiotherapy combined with immunotherapy: the dawn of cancer treatment. Signal Transduct Target Ther. 2022;7(1):258. 35906199 10.1038/s41392-022-01102-y PMC9338328

